# Supplementary material for: Overcoming global inequality is critical for land-based mitigation in line with the Paris Agreement
Source: Nat Commun. 2022 Dec 2;13:7453. doi: 10.1038/s41467-022-35114-7 (PMC9718475; doi:10.1038/s41467-022-35114-7)
Supplement: Supplementary file 1 — Supplementary Information [file 41467_2022_35114_MOESM1_ESM.pdf]

Overcoming global inequality is critical for land-based mitigation in line with the Paris Agreement

Supplementary Information (SI)

Florian Humpenöder[1*], Alexander Popp[1], Carl-Friedrich Schleussner[2,3], Anton Orlov[4], Michael Gregory Windisch[1,3], Inga Menke[2,3], Julia Pongratz[5,6], Felix Havermann[5], Wim Thiery[7], Fei Luo[8,9], Patrick v. Jeetze[1,3], Jan Philipp Dietrich[1], Hermann Lotze-Campen[1,3], Isabelle Weindl[1], Quentin Lejeune[2]

[1]Potsdam Institute for Climate Impact Research (PIK), Member of the Leibniz Association, Potsdam, Germany.
[2]Climate Analytics (CA), Berlin, Germany.
[3]Humboldt University of Berlin, Berlin, Germany.
[4]CICERO, Oslo, Norway.
[5]Ludwig-Maximilians-University (LMU) Munich, Munich, Germany.
[6]Max Planck Institute for Meteorology, Hamburg, Germany.
[7]Vrije Universiteit Brussel, Brussels, Belgium.
[8]Institute for Environmental Studies, Vrije Universiteit Amsterdam, Amsterdam, Netherlands
[9]Royal Netherlands Meteorological Institute (KNMI), De Bilt, Netherlands

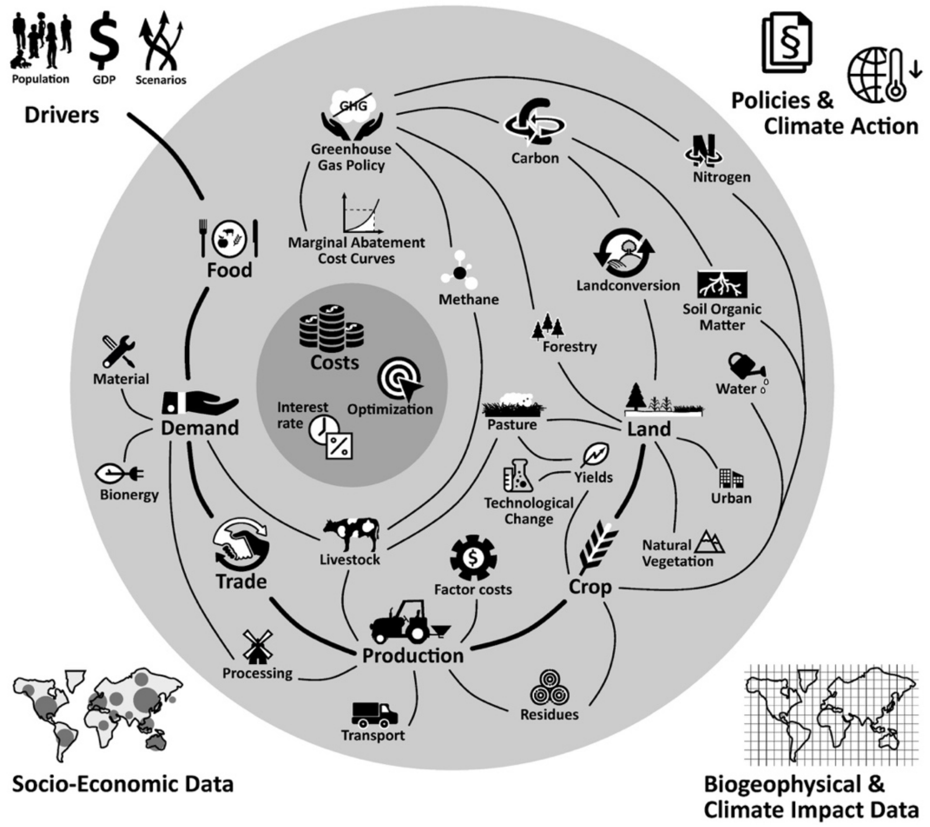

Figure S1: MAgPIE 4 framework simplified modular structure and module interactions, reproduced from Dietrich, J. P., et al. MAgPIE 4 – a modular open-source framework for modeling global land systems, Geosci. Model Dev., 12, 1299–1317, https://doi.org/10.5194/gmd-12-1299-2019, CC-BY-4.0.

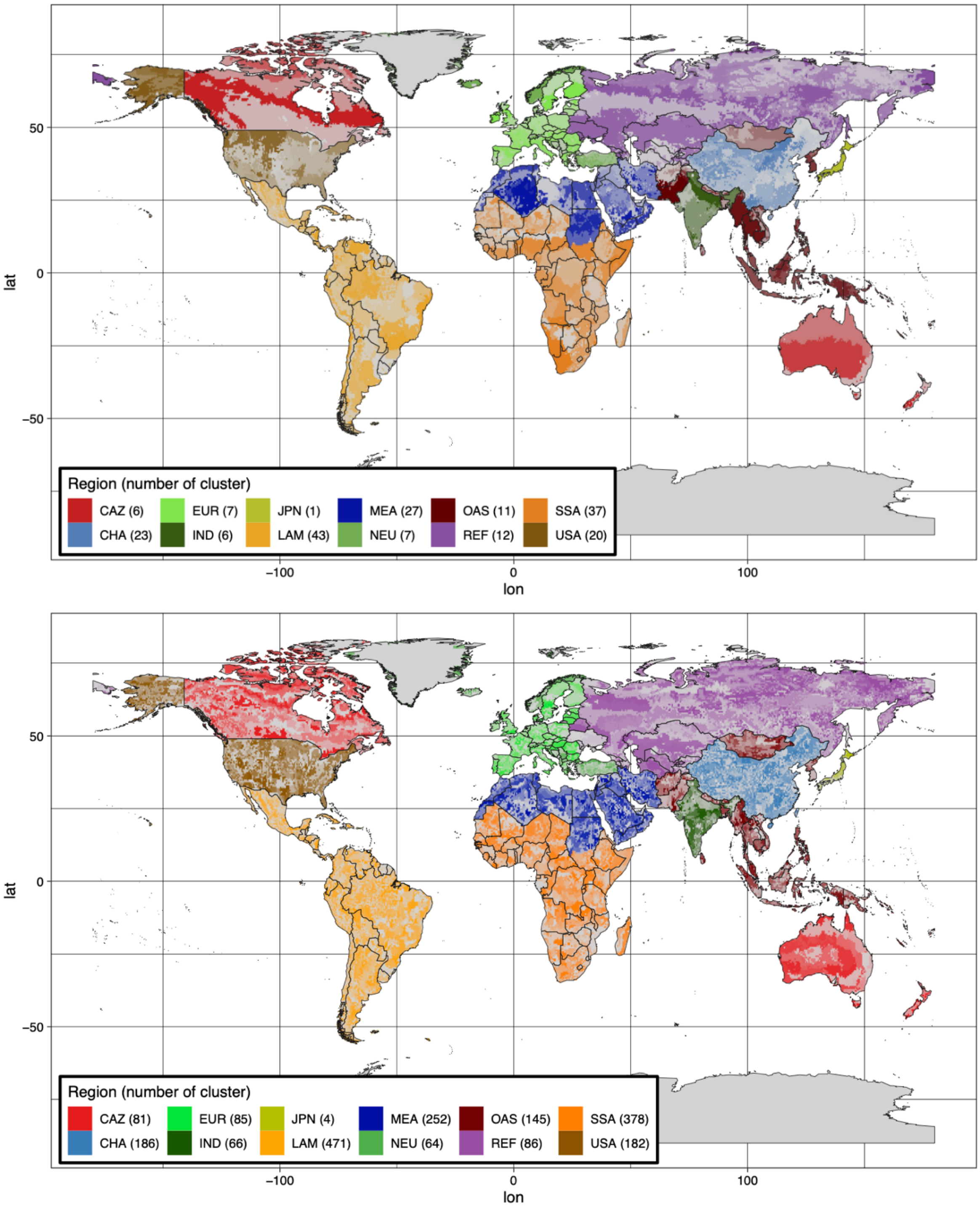

Figure S2: Map of MAgPIE regions and associated spatial simulation units. Top: Simulation units used for global optimization (200 in total). Bottom: Simulation units used for parallel optimization with exogenous trade patterns (2000 in total). Regional definitions: CAZ (Canada, Australia, and New Zealand; CHA (China); EUR (European Union); IND (India); JPN (Japan); LAM (Latin America); MEA (Middle East and north Africa); NEU (non-EU member states); OAS (other Asia); REF (reforming countries); SSA (Sub-Saharan Africa); USA (United States). The country map is based on the object " wrld_simpl" in the R package "maptools".

SDG	Indicator and Unit	Definition	SDG target 2030/2050	Source / Comment
SDG 2	prevalence of underweight	Number of adults with body mass index (BMI) < 18.5 and children with < -2 standard deviations (SD) from reference BMI	0	Soergel et al 2021[7] Underweight is covered by SDG indicator 2.1.1
SDG 3	prevalence of obesity	Number of adults with BMI > 30 and children (aged 0-14) with > 2 SD from reference BMI	-	Soergel et al 2021[7] Obesity is covered by SDG Target 2.2
SDG 6	Agricultural water use (km^3 yr^{-1})	Water use for irrigation and other agricultural purposes	-	Bonsch et al 2014[55]
SDG 13	CO_2 emissions and removals from land-use change and management (Gt CO_2 eq yr^{-1})	Annual net CO_2 emissions accounting for carbon losses through deforestation, conversion of non-forest ecosystems (including peatlands) and wood harvest as well as for carbon gains from re/afforestation, timber plantations, natural regrowth and storage in wood products. The calculation of annual net CO_2 emissions is based on carbon stock changes between time steps (except for peatlands).	-	Humpenöder et al 2020[45] and 2022[56] Mishra et al 2021[40] To avoid that our results are biased by the values of single years (stock-flow problem), we calculate in a post-processing step an average value by applying a low-pass filter function that distributes values over time, while making sure that the time integral remains the same.
SDG 13	CH_4 emissions from agriculture (Gt CO_2 eq yr^{-1}) IPCC AR6 GWP100 factor of 27	CH_4 emissions from enteric fermentation, animal waste management and rice cultivation, estimated based on feed demand, manure, and rice cultivation area, respectively.	-	Popp et al 2010[44] Stevanović et al 2017[42]
SDG 13	N_2O emissions from agriculture (Gt CO_2 eq yr^{-1}) IPCC AR6 GWP100 factor of 273	N_2O emissions from agricultural soils (fertilizer application) and animal waste management, estimated based on nitrogen budgets for croplands, pastures and the livestock sector.	-	Bodirsky et al 2014[43] Stevanović et al 2017[42]
SDG 15	Forest area without plantations (Change in Mha compared to 2020)	Change of primary forest, secondary forest, and re/afforested areas, but excluding timber plantations.	Conserve and restore terrestrial ecosystem	
SDG 15	Nitrogen fixation (Mt N yr^{-1})	Nitrogen fixation is a proxy for nitrogen losses to the environment Nitrogen inputs on cropland via industrial (e.g. production of inorganic fertilizers) and intentional biological fixation are calculated based on a nitrogen budget approach	62 Mt N yr^{-1}	Nitrogen fixation as an indicator follows Soergel et al 2021[7] and van Vuuren et al 2022[26]. Quantitative target from Steffen et al 2015[25].

Table S1: Overview of indicators assessed in this study. All indicators have been derived from the MAgPIE model and mapped to corresponding Sustainable Development Goals (SDGs). SDG 2: end hunger. SDG 3 health. SDG 6: clean water and sanitation. SDG 13: climate action. SDG 15: life on land.

	2020	*Inequality* - 2030	Sustainability - 2030
**OECD90+EU**	151	566	568
**ASIA**	38	38	356
**LAM**	83	83	822
**ROW**	17	17	404
**SSA**	63	63	272
**World**	352	767	2522

Table S2: Protected area in Mha in forest and non-forest natural land.

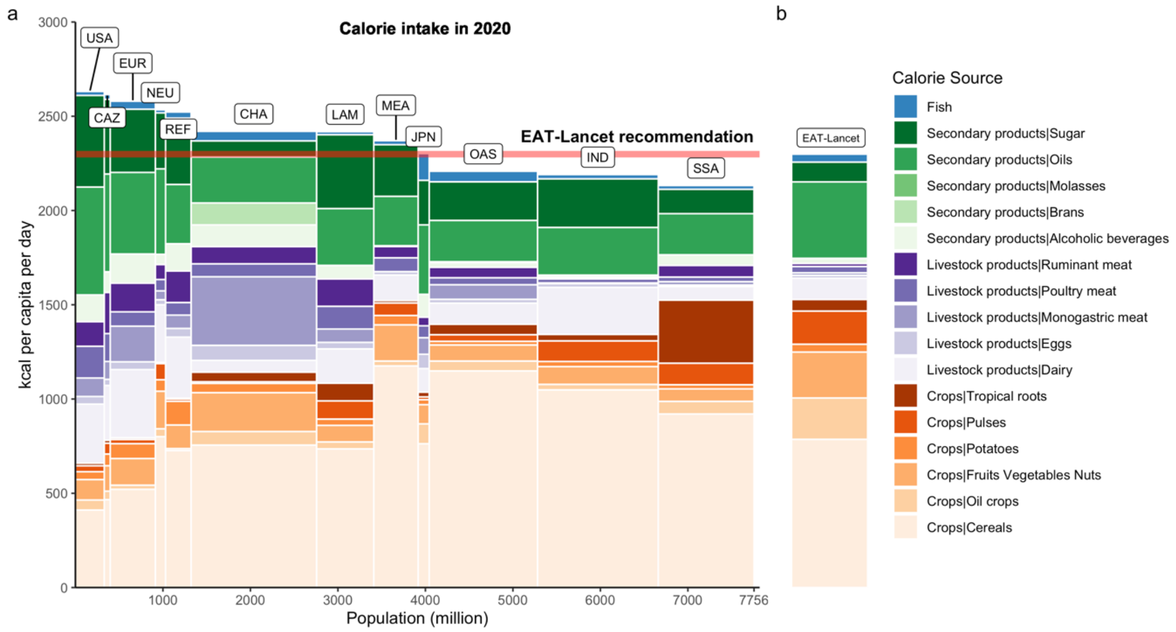

Figure S3: Per-capita calorie intake. a) shows regional data for 2020. The height of each rectangle shows per-capita kcal intake, the width shows the population of the region, so that the area of the rectangles refers to the total calorie intake for each region. b) shows EAT-Lancet recommendations (planetary health diet), aggregated to global level. In the Global-Sustainability scenario, all regions converge towards the EAT-Lancet recommendation by 2050.

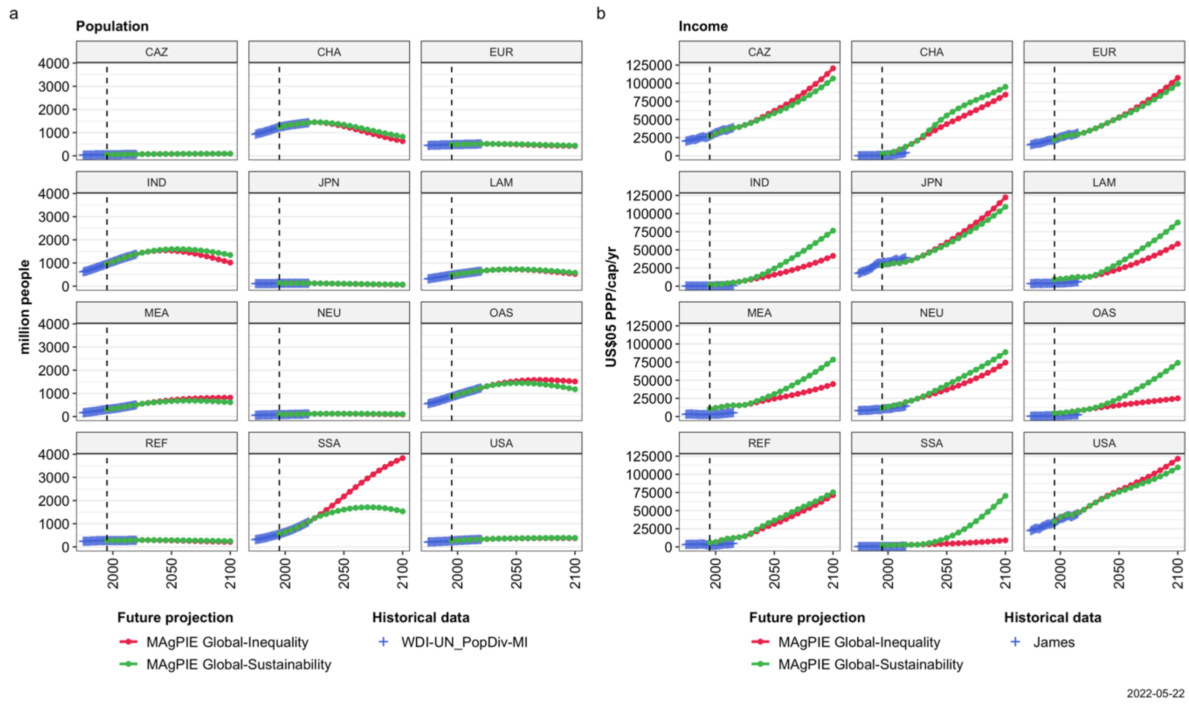

Figure S4: Population and Income. Data is shown at regional level for the two main scenarios. Global-Inequality is based on SSP4. Global-Sustainability is based on SSP1. a) shows regional projections of population based on KC and Lutz (https://doi.org/10.1016/j.gloenvcha.2014.06.004). Global-Inequality is based on SSP4. Global-Sustainability is based on SSP1. b) shows regional projections of income based on Dellink et al (https://doi.org/10.1016/j.gloenvcha.2015.06.004). Historical data for comparison from World Bank World Development Indicators (WDI) (https://wdi.worldbank.org/) and James et al. 2012 (https://doi.org/10.1186/1478-7954-10-12).The historical data has been processed using the pik-piam/mrvalidation R package (https://doi.org/10.5281/zenodo.4317826)

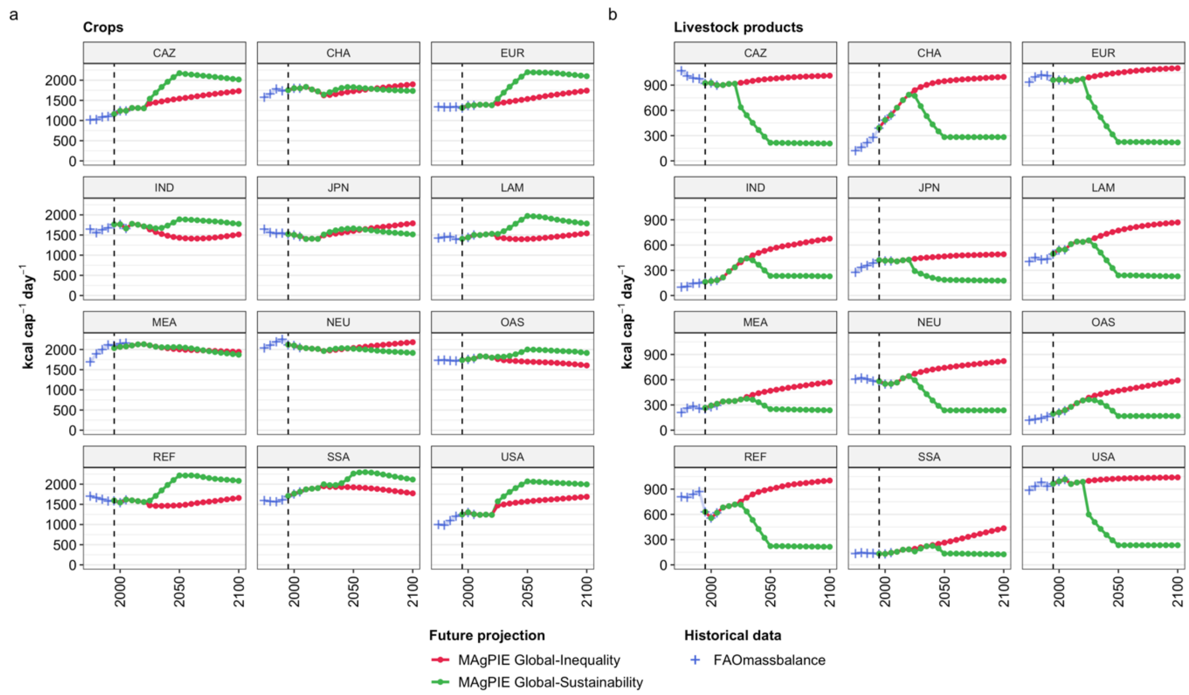

Figure S5: Per-capita calorie supply. Data is shown at regional level for the two main scenarios. a) shows crops. b) shows livestock products. Historical data from FAO (https://www.fao.org/faostat). The historical data has been processed using the pik-piam/mrvalidation R package (https://doi.org/10.5281/zenodo.4317826).

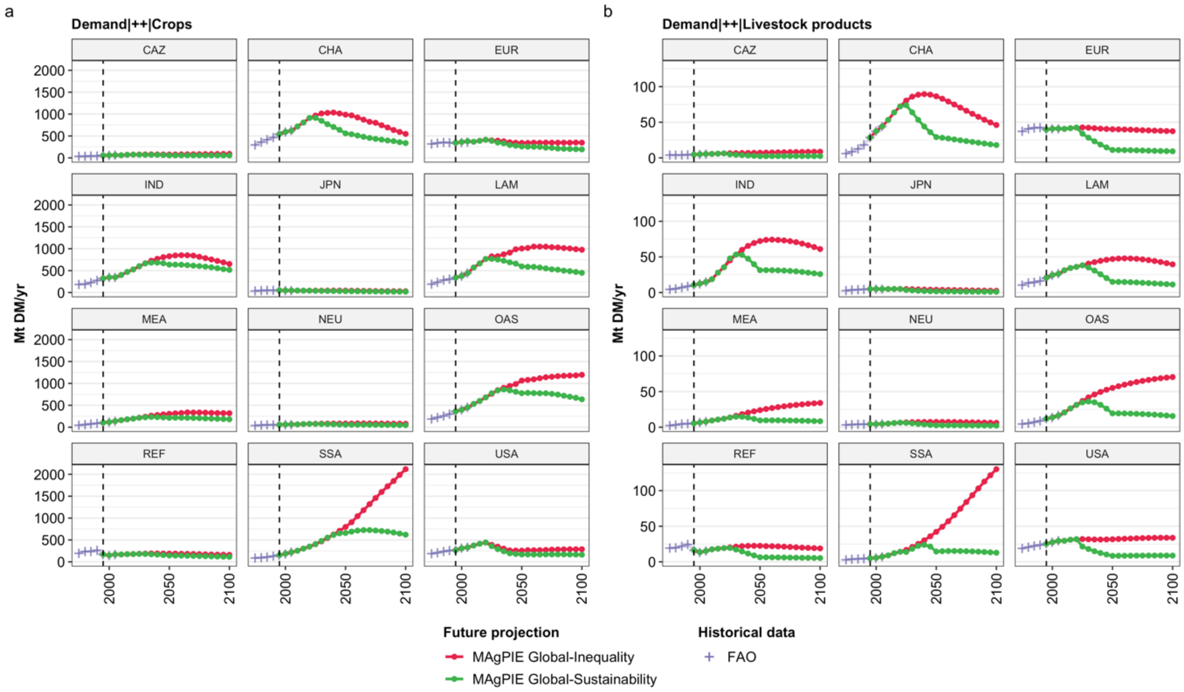

Figure S6: Total agricultural demand. Data is shown at regional level for the two main scenarios. a) shows crops (including food and feed. b) shows livestock products. Historical data for comparison from FAO (https://www.fao.org/faostat). The historical data has been processed using the pik-piam/mrvalidation R package (https://doi.org/10.5281/zenodo.4317826).

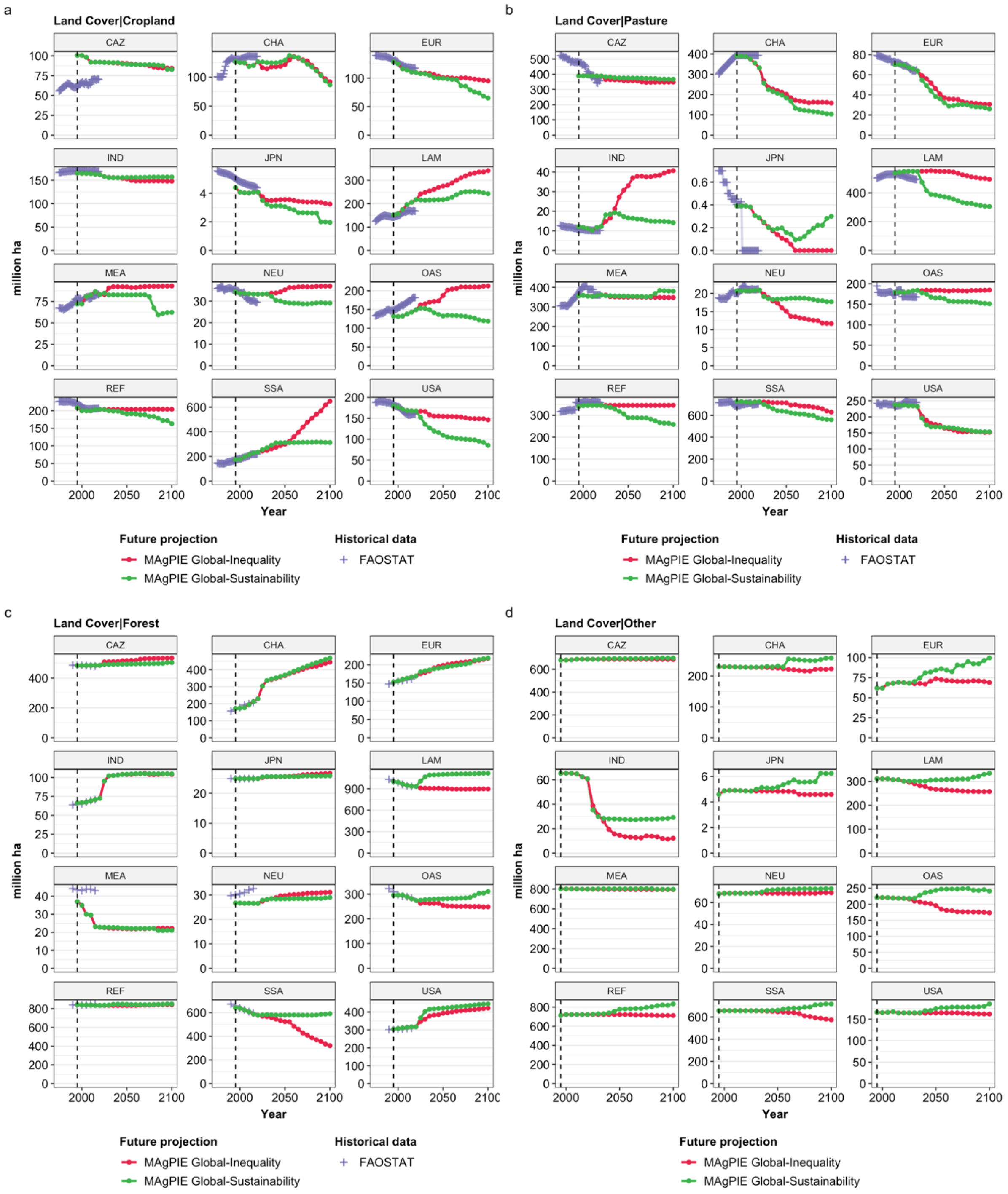

Figure S7: Validation of main land classes. Data is shown at regional level for the two main scenarios. a) shows cropland. b) shows pasture. c) shows forest. d) shows other natural land. Historical data for comparison from FAO (https://www.fao.org/faostat).The historical data has been processed using the pik-piam/mrvalidation R package (https://doi.org/10.5281/zenodo.4317826).

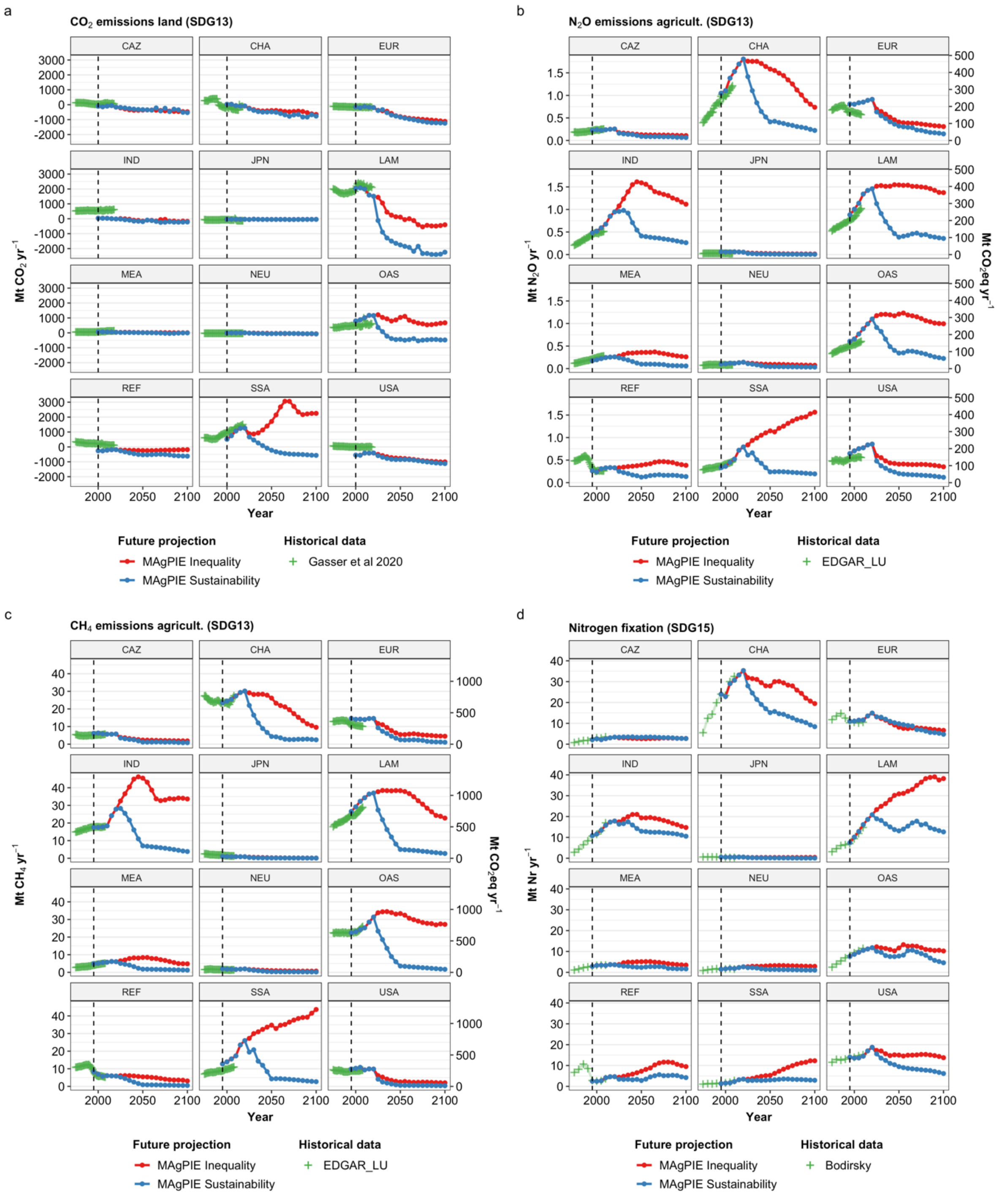

Figure S8: Validation of environmental indicators: GHG emissions and nitrogen fixation. Data is shown at regional level for the two main scenarios. a) shows net CO_2 emissions from land-use change and management. b) shows N_2O emissions from agriculture. c) shows CH_4 emissions from agriculture. d) shows nitrogen fixation. For the conversion of N_2O and CH_4 emissions into CO_2 equivalents (right axis) we used IPCC AR6 GWP100 factors of 273 and 27, respectively. Historical data for comparison from Gasser et al 2020 (https://doi.org/10.5194/bg-17-4075-2020), the EDGAR emissions database version 4.2 (https://doi.org/10.2904/EDGARv4.2) and Bodirsky et al 2014 (https://doi.org/10.1038/ncomms4858). The historical data has been processed using the pik-piam/mrvalidation R package (https://doi.org/10.5281/zenodo.4317826).

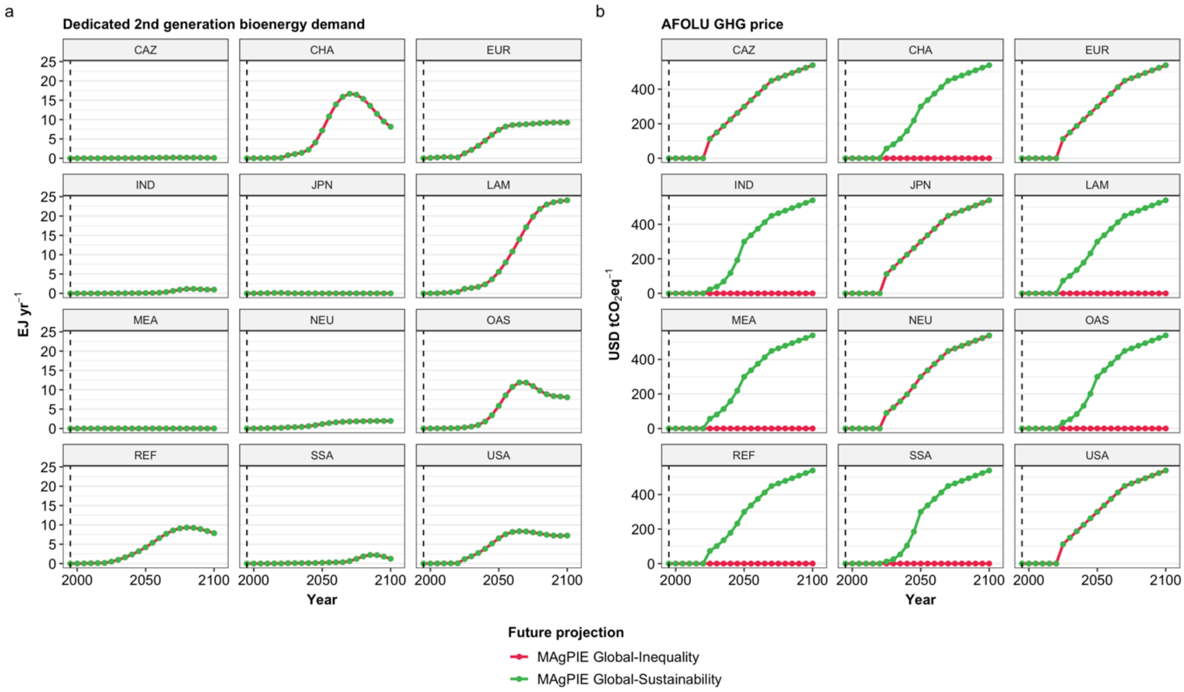

Figure S9: Assumptions for land-based mitigation. Data is shown at regional level for the two main scenarios. a) shows dedicated 2nd generation bioenergy demand. b) shows AFOLU GHG prices. Data is taken from the SDP-1.5C scenario (RCP1.9) in Soergel et al 2021 (https://doi.org/10.1038/s41558-021-01098-3) In the Inequality scenario, GHG prices are active only in those regions that belong to OECD90+EU (Table 2).

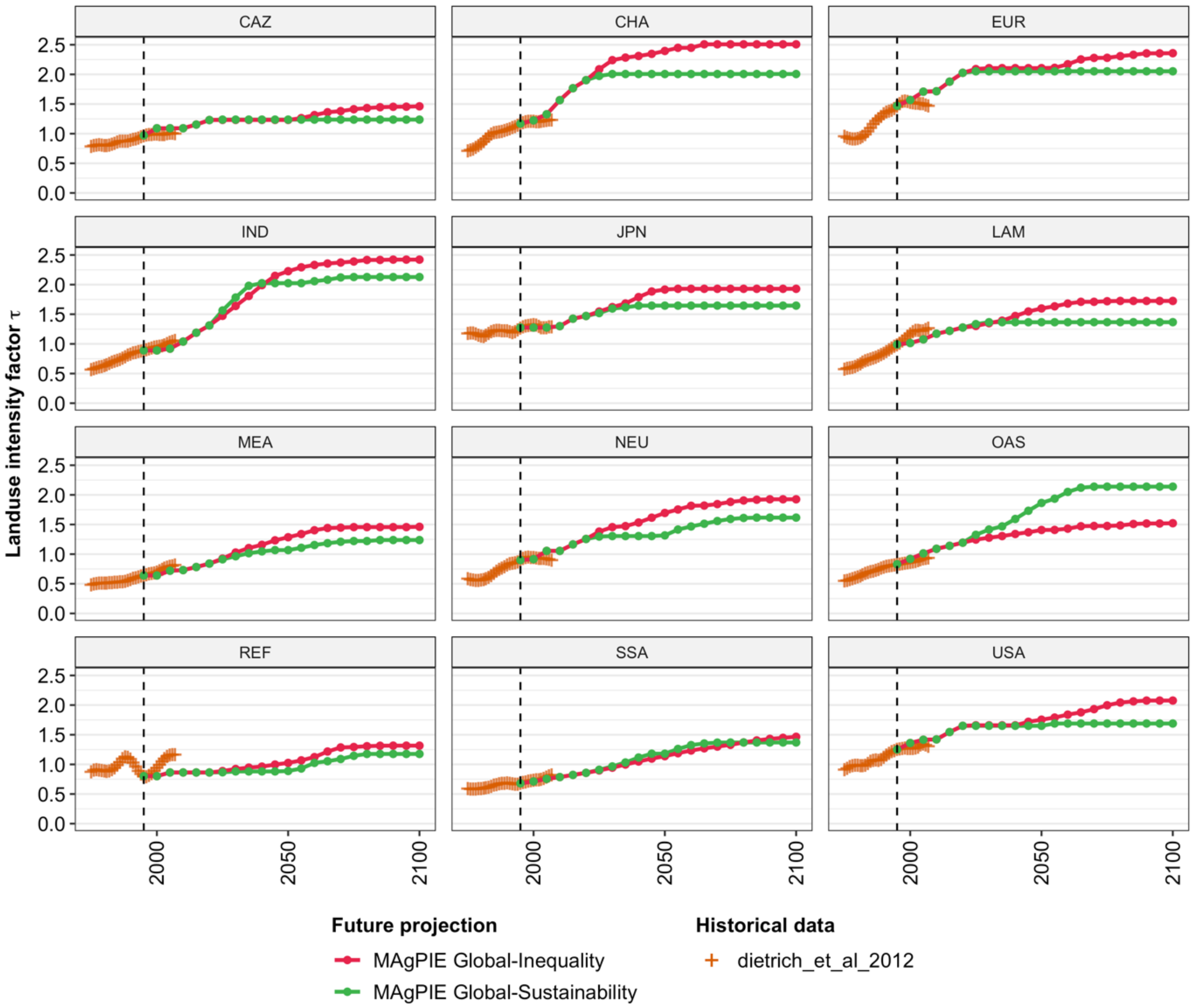

Figure S10: Land-use intensity factor τ. The τ factor reflects the degree of crop yield amplification caused by human activities. A duplication of τ implies a doubling of crop yields under fixed environmental conditions. Data is shown at regional level for the two main scenarios.
Historical data for comparison from Dietrich et al 2012 (https://doi.org/10.1016/j.ecolmodel.2012.03.002). The historical data has been processed using the pik-piam/mrvalidation R package (https://doi.org/10.5281/zenodo.4317826)

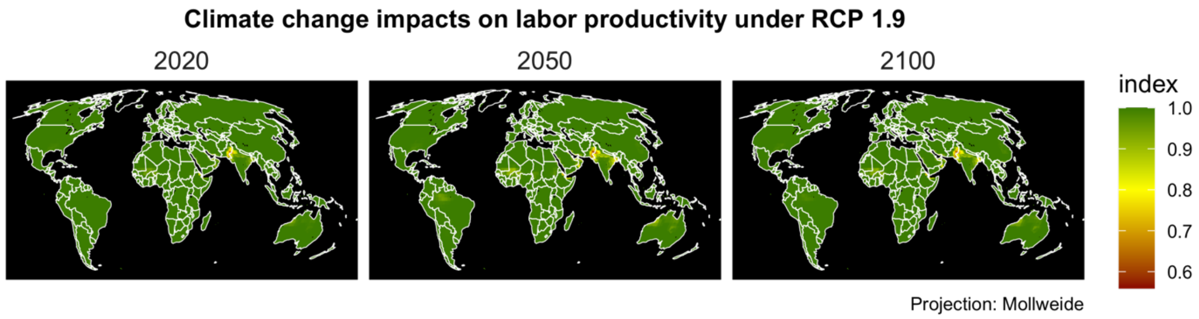

Figure S11: Climate change impacts on labor productivity under RCP 1.9. Data is shown as index relative to full labor productivity (1). Heat-induced impacts on labor productivity for RCP 1.9 (ISO metric at 400 watt work intensity) have been calculated using the methodology described in Orlov et al 2021 (https://doi.org/10.1007/s41885-021-00091-6), based on data from the LAMACLIMA project. The country map has been created with the function "ne_countries" in the R package "rnaturalearth".
