# Supplementary material for: Overcoming global inequality is critical for land-based mitigation in line with the Paris Agreement
Source: Nat Commun. 2022 Dec 2;13:7453. doi: 10.1038/s41467-022-35114-7 (PMC9718475; doi:10.1038/s41467-022-35114-7)
Supplement: Supplementary file 3 — Description of Additional Supplementary Files [file 41467_2022_35114_MOESM3_ESM.pdf]

File name: Supplementary Data 1

Description: includes numerical scenario results as well as scripts for figure generation.

File name: Supplementary Data 2

Description: includes raw data for all figures in the manuscript.
